# On the nose: nasal neurostimulation as a technology countermeasure for sinonasal congestion in astronauts

**DOI:** 10.3389/fphys.2025.1536496

**Published:** 2025-02-14

**Authors:** Timon Ax, Philipp H. Zimmermann, Tomas L. Bothe, Karen Barchetti, Cintia S. de Paiva, Francesc March de Ribot, Slade O. Jensen, Thomas J. Millar

**Affiliations:** ^1^ School of Medicine, Western Sydney University, Sydney, NSW, Australia; ^2^ Department of Ophthalmology, Saarland University Medical Center, Homburg/Saar, Germany; ^3^ Department of Otorhinolaryngology, Head and Neck Surgery, Medical Faculty, University of Cologne, Cologne, Germany; ^4^ Institute of Physiology, Center for Space Medicine and Extreme Environments Berlin, Charité – Universitätsmedizin Berlin, Berlin, Germany; ^5^ Université Paris Cité, Faculté de Pharmacie, MTCI ED 563, Paris, France; ^6^ INSERM UMRS 970 Paris Centre de Recherche Cardiovasculaire (PARCC), Paris, France; ^7^ Ocular Surface Center, Department of Ophthalmology, Baylor College of Medicine, Cullen Eye Institute, Houston, TX, United States; ^8^ Department of Ophthalmology, Otago University, Dunedin, New Zealand; ^9^ Department of Ophthalmology, Girona University, Girona, Spain; ^10^ Antimicrobial Resistance and Mobile Elements Group, Ingham Institute of Applied Medical Research, Sydney, NSW, Australia; ^11^ Beyond 700 Pty Ltd, Sydney, NSW, Australia

**Keywords:** sinus pain, nasal congestion, microgravity, countermeasure, neurostimulation, sinusitis, human spaceflight

## Abstract

Human spaceflight subjects the body to numerous and unique challenges. Astronauts frequently report a sense of sinonasal congestion upon entering microgravity for which the exact pathomechanisms are unknown. However, cephalad fluid shift seems to be its primary cause, with CO_2_ levels and environmental irritants playing ancillary roles. Current management focuses on pharmacotherapy comprising oral and nasal decongestants and antihistamines. These are among the most commonly used treatments in astronauts. With longer and more distant space missions on the horizon, there is a need for efficacious and payload-sparing non-pharmacological interventions. Neurostimulation is a promising countermeasure technology for many ailments on Earth. In this paper, we explore the risk factors and current treatment modalities for sinonasal congestion in astronauts, highlight the limitations of existing approaches, and argue for why neurostimulation should be considered.

## 1 Introduction

The sinonasal system consists of the air-filled nasal cavity, including the turbinates, and the adjacent sinuses, separated by the nasal septum. Its mucosal lining, rich in blood vessels, glands, and nerve endings, supports functions such as smelling, humidifying, cleaning, and warming inhaled air, while also providing immune defense (Elad et al., 2008; Sahin-Yilmaz and Naclerio, 2011). The nasal cycle, alternating congestion and decongestion between sides, helps maintain nasal functions but can be disrupted by nasal congestion, which also affects adjacent organs such as the eyes and ears (Pendolino et al., 2018; Susaman et al., 2021).

In-flight nasal congestion and sinonasal symptoms (facial pressure and pain or “sinus pain”) were reported by 62% of space shuttle crew members during postflight medical debriefings (Clément, 2011; Khan et al., 2024). Congestion was the most common otorhinolaryngological complaint among ISS astronauts and one of the most frequent complaints in general (Alexander, 2021). Thus, NASA considers nasal congestion highly likely to occur during any space mission (NASA, 2016).

Sinonasal congestion, like dry eye disease, is more of a nuisance than an immediate medical risk (Ax et al., 2023). However, it can interfere with mission tasks, thereby compromising productivity, cause fluid loss from the body through mouth breathing, and change smell and taste (Rudmik et al., 2014; Lane et al., 2016; Hummel et al., 2017; Marshburn et al., 2019). Nasal congestion increases the likelihood of barotrauma in situations of environmental pressure changes such as during extravehicular activity (Iannella et al., 2017; Pilmanis and Clark, 2019; Chen et al., 2023). Moreover, it can exacerbate the already highly prevalent sleep issues in orbit (Albornoz-Miranda et al., 2023). Over time, mucosal edema might impair the nasal cycle, cause eustachian tube dysfunction and reduce ventilation of the paranasal sinuses, thereby increasing infection risk (Marshburn et al., 2019; Macias et al., 2020; Susaman et al., 2021). An unexpected but likely consequence of mucosal swelling could be tear dysfunction through decreased nasal tear drainage and tear production; nasal breathing contributes around 30% to basal tear secretion (Gupta et al., 1997; Ax et al., 2023). Nasal congestion in combination with elevated CO_2_ levels may also contribute to the frequent headaches observed during spaceflight (Law et al., 2014; Kazaz et al., 2021).

## 2 Potential mechanisms and risk factors

### 2.1 Cephalad fluid shift

Microgravity causes ∼2L of fluid to move towards the upper body and head of astronauts within the first 6–10 h (Thornton et al., 1987). This phenomenon is called cephalad fluid shift (CFS), typified by facial puffiness and bird legs (Thornton et al., 1974). CFS is the major contributing factor to sinonasal congestion in astronauts (Hargens and Richardson, 2009; Marshall-Goebel et al., 2019; Stenger and Macias, 2020). CFS-related congestion is likely to occur in the abundant spongy tissue filled with venous sinusoids in the nasal mucosa (Burnham, 1941; Ng et al., 1999). These tissues have limited ways of regulating their microcirculation during CFS and therefore experience fluid extravasation (Aratow et al., 1991; Parazynski et al., 1991). On-orbit examination shows increased erythema and edema of the nasal mucosa (Harris et al., 1997). Periorbital puffiness, facial edema and thickening of the eyelids last to varying degrees for the entire duration of microgravity exposure making persistent intranasal swelling likely (Schneider et al., 2016; Hamilton, 2019; Karlin et al., 2021).

The effects of CFS are difficult to study upon return to Earth because they disappear. Nevertheless, magnetic resonance imaging showed increased mastoid effusions after ISS missions although there were no changes in the paranasal sinuses (Inglesby et al., 2020). Asymptomatic mastoid effusions are also known to occur in supine patients (head-down bed rest, intensive care unit patients) making a strong case for CFS being their primary cause (Huyett et al., 2017; Lecheler et al., 2021). Remarkably, facial tissue thickness was below control values immediately on return to Earth reaching baseline values after 4 days (Kirsch et al., 1993).

### 2.2 CO_2_ levels

CO_2_ levels are at least 10 times higher on the ISS than on Earth (Law et al., 2014; Lee et al., 2020). CO_2_ is a potent vasodilator and may lead to further engorgement of the nasal mucosal vessels (Ito et al., 2003).

This factor might partially explain why sinonasal symptoms persist over many months even though facial puffiness redistributes a few days after entering microgravity (Kirsch et al., 1993; Cole et al., 2019). CO_2_ has also been implicated in dry eye disease and headaches in astronauts (Law et al., 2014; Sampige et al., 2024).

However, while similarly high CO_2_ levels are found in submarines, decongestant use in submariners is ∼150 times lower than in astronauts suggesting that CO_2_ might just be a minor contributor to sinonasal congestion in microgravity (Wotring, 2015).

Enigmatically, CO_2_ applied directly to the nasal mucosa is used to treat both nasal congestion and migraine headaches likely by suppressing neuropeptide release from the trigeminal nerves (Hurst, 1931; Casale et al., 2008; Spierings, 2024).

### 2.3 Environmental irritants

Despite extensive screening of astronauts for allergies, allergic symptoms are prevalent and contribute to sinonasal congestion (Wotring, 2015). Most likely, this is caused by increased exposure to bioaerosols as dust does not settle in microgravity and spacecraft are closed environments in which allergens and irritants accumulate, and microbe growth is promoted (Oubre et al., 2016; Jahn et al., 2021).

Even in the absence of a specific allergy, nasal mucosa might become hyperreactive to irritants and allergens in space because of immune system alterations (Crucian et al., 2013; Torun et al., 2021). Changes to the nasal microbiome might further contribute to mucosal inflammation (Salzano et al., 2018). Nasal toxicity of extraterrestrial dust should also be considered for upcoming Moon and Mars missions (Miranda et al., 2023). Lunar dust has already demonstrated its irritative properties during the Apollo missions (Hardison et al., 2023), and Martian dust contains dust contains irritant, reactive perchlorates (Davila et al., 2013; Crotts, 2014).

## 3 Countermeasures

### 3.1 Pharmacological countermeasures

Astronauts take decongestant medication and antihistamines to combat sinonasal symptoms. The use of antibiotics is uncommon since acute respiratory infection and consequent bacterial sinusitis are very rare due to strict preflight screening and quarantine regimens (Alexander, 2021; Vernikos, 2022). Decongestants mimic sympathetic activation leading to vasoconstriction and reduced mucosal swelling (Johnson and Hricik, 1993), while antihistamines block the vasodilative effect of histamine at the H1 receptor (Ashina et al., 2015).

Decongestants are the most common medication used chronically (>7 days) by ISS astronauts, and the third most used in the acute context. Overall, 55% of astronauts reported use of decongestant medication with 2.4 medication uses per crew member for ISS missions (Wotring, 2015). Monitoring medication use relies on astronauts self-reporting during postflight debriefings or flight physician notes from private medical conferences. Thus, actual decongestant use is likely to be higher due to underreporting (Wotring, 2015; Blue et al., 2019).

Pharmacotherapy during spaceflight has assumed that pharmacokinetics and pharmacodynamics are comparable to those on Earth (Grover and Pathak, 2020; Barchetti et al., 2024). This may not be completely true, given the different outcomes reported by astronauts. Regarding decongestants, 21% of astronauts report them being very effective with the remainder stating “somewhat effective” (39%), “ineffective” (2%) or “unknown” (37%) due to lack of information (Blue et al., 2019).

Topical decongestants come in the form of drops and sprays. Nasal drop application in microgravity is problematic because a globule of fluid must be wicked into the nose instead of “dropping” it. These globules risk resource waste and overdose because they are three to six times the size of a regular drop (Mader et al., 2019). Long-term use could lead to dependency and drug-induced rhinitis inherent with topical decongestants (Varghese et al., 2010).

Contact of the dropper bottle with the mucosa prohibits sharing among crew members due to contamination (Aydin et al., 2007). Nasal sprays have the additional risk of (bio)aerosol generation.

Systemic drugs are easier to use but more likely than topical ones to have side effects that involve other organs, such as exacerbating dry eye symptoms through their anticholinergic effects (Gomes et al., 2017; Unsal et al., 2018). Payload requirements, finite supplies and use-by dates limit medication availability in space. Despite the presence of a pharmacy onboard the ISS, the awareness by astronauts that medications are a scarce resource leads to a reluctance to use them even when potentially beneficial (Barchetti et al., 2024).

### 3.2 Non-pharmacological and environmental countermeasures

Non-pharmacological solutions remove the restrictions associated with medication use. To counter CFS, a low-tech solution such as *Braslet* occlusion cuffs sequesters fluid in the lower extremities and reduces facial puffiness (Hamilton et al., 2012). Whether this also ameliorates symptoms is unclear (Schneider et al., 2016). Lower body negative pressure and artificial gravity are other alternatives but are technically more challenging (Clement et al., 2015; Hamilton, 2019).

CO_2_-related symptoms might be reduced by more effective approaches to monitor and scrub the cabin atmosphere of excess CO_2_ (Georgescu et al., 2020; Georgescu et al., 2021). Similarly, better air filtration and cabin hygiene could reduce bioaerosols, leading to fewer allergic symptoms (Haines et al., 2019; Marshburn et al., 2019).

### 3.3 Neurostimulation

Engorgement of the nasal vasculature through CFS and other factors (CO_2_, environmental irritants) is the main cause of sinonasal symptoms in astronauts (Stenger and Macias, 2020). Nasal vessels are modulated by nerve fibers of the autonomic nervous system (ANS) (Baraniuk and Merck, 2009; Kahana-Zweig et al., 2016) whereby sympathetic vasoconstriction chiefly determines nasal patency on Earth (Lung, 1995; Susaman et al., 2021). The ANS also partly mediates mucociliary clearance, a process essential for the removal of mucus and irritants, which is potentially impaired in space (Beule, 2010; Prisk, 2019; Smith et al., 2024). Thus, dysfunction of the ANS contributes causally to sinonasal congestion (Yao et al., 2018).

In astronauts, targeted sympathetic activation might counteract both CFS and CO_2_-related vasodilation in the nasal mucosa (Shusterman et al., 2023). Neurostimulation is a technique that offers therapy by targeted modulation of neural activity. It is widely used in treating conditions as diverse as epilepsy, diabetes, and chronic pain (Errico, 2018; Mehta et al., 2018; Stanton-Hicks, 2018).

On Earth, several neurostimulation methods have been introduced to relieve nasal congestion in allergic and chronic rhinosinusitis patients (Phillips et al., 2022; Shusterman et al., 2023). Similar methods are being explored for treating dry eye disease (Mittal et al., 2021). In both cases, the target nerve is the anterior branch of the ethmoidal nerve, itself part of the trigeminal nerve (Dieckmann et al., 2019; Li et al., 2020). This nerve can be accessed intra-nasally through electrical, mechanical, and pharmaceutical stimulation as well as extra-nasally through mechanical and magnetic stimulation (Table 1).

**TABLE 1:** Comparison of Nasal Neurostimulation modalities/types (Blue: ocular; grey: nasal; violet: both).

Modality	Device/Drug	Advantages	Disadvantages	References
Mechanical/Percussive	iTear100 (Olympic Ophthalmic, United States)	-Effective for both sinonasal symptoms and dry eye -Can be shared -Rechargeable -Small -No consumables -Quick (30 s) -Activity is logged in companion app	-Feels tickly upon first application -Local skin irritation	Ji et al. (2020), Shusterman et al. (2023)
	Chordate System S101 (Chordate Medical AB, Sweden)	-Possible long-term effect (up to 1 year post-treatment)	-Bulky device -Consumables (catheter-connected latex balloon) -Sneezing is a common side effect -Long treatment duration (10 min in each nasal cavity)	Juto and Axelsson (2014), Sainio et al. (2023)
	SONU (Sound Health Systems, United States)	-Personalized through smartphone app -Can be shared -No consumables -Rechargeable -Hands-free operation	-Long treatment duration (20 min)	Khanwalkar et al. (2022), Luong et al. (2024)
	SinuSonic (Healthy Humming, United States)	-Small -Rechargeable -Relatively short treatment duration (3 min)	-Consumables (silicone nosepiece) -Hygiene concerns (aerosol generation)	Cairns and Bogan (2019), Soler et al. (2020)
Electrical	TrueTear (Allergan, United States)^a^	-Proven efficacy in dry eye disease -Rechargeable -Small	-Consumables (hydrogel tips) -Risk of injury b/c invasive -Effect on sinonasal symptoms unknown	Gumus et al. (2017), Watson et al. (2017), Farhangi et al. (2019), Pondelis et al. (2020)
	ClearUp (Tivic Health, USA)	-Specifically designed to treat sinonasal discomfort -External device -No consumables -Small -Rechargeable -Can be shared	-Relatively long treatment duration (5 min)	Goldsobel et al. (2019), Maul et al. (2019)
Magnetic	Viveye OMN (EpiTech, Israel)	-External device -Painless procedure	-Bulky device -Few human data -Effect on sinonasal symptoms unknown	Ben-Eli et al. (2024)
Pharmacological	Tyrvaya nasal spray (Varenicline; Oyster Point Pharma, United States)	-Effective for dry eye disease -Quick application (seconds)	-Sneezing is a very common side effect -Bioaerosol generation -Cannot be shared -Limited shelf life -Effect on sinonasal symptoms unknown	Wirta et al. (2022)

^a^ No longer commercially available.

Proven terrestrial efficacy does not automatically deem an approach suitable for use in space. Some neurostimulation devices are too bulky whereas others need consumables to function (Table 1). Extra-nasal devices have a smaller injury risk compared to intra-nasal (invasive) devices. Additionally, intra-nasal devices trigger sneezing as a side effect more frequently which might expedite the spread of disease vectors throughout the spacecraft cabin (Mermel, 2013; Wirta et al., 2022).

Pharmacological neurostimulation comes with all described constraints associated with pharmacotherapy in space and thus offers no clear advantages over drugs already in use.

In our view, there are currently three devices which can be considered for use in astronauts (Table 1).

1. iTear100 is an extranasal mechanical neurostimulator that has proven effective for treating both ocular and sinonasal symptoms.
2. SONU is a vibrational headband that gets programmed to match the natural resonant frequency of the sinonasal cavity of the individual.
3. ClearUp uses extranasal electrical stimulation and is specifically approved to treat sinonasal symptoms.

The advantages of these devices are that they are small in size, rechargeable, lack consumables, and have minimal side effects. A single device can be utilized by multiple crewmembers and use can be logged automatically to provide accurate data on use frequency (Wotring and Smith, 2020). However, there are still many unknowns associated with their appropriate application: ideal modality (electrical versus mechanical), intensity and frequency of application, duration and size of treatment effect as well as possible adaptation to the stimulus remain to be determined in astronauts. Device settings may also be tailored to the individual astronaut by developing treatment protocols (e.g., duration, intensity, frequency of stimulation) based on crewmembers’ specific physiology and needs (Denison and Morrell, 2022).

## 4 Discussion and conclusion

Sinonasal congestion is very common in astronauts. Mild cases may impact astronaut wellbeing and productivity, while severe cases could substantially hinder the execution of mission-critical tasks. Nasal neurostimulation has the potential to provide a safe and effective non-pharmacological treatment option for sinonasal congestion in astronauts, thus overcoming the limitations of using pharmaceuticals in space. The apparently common practice among astronauts of long-term decongestant use is of particular concern (Wotring, 2015) and could in itself be a significant factor for long-term nasal congestion since continued use decreases responsiveness to subsequent decongestion efforts (Varghese et al., 2010). Neurostimulation is attractive because it offers an avenue to reduce or even replace decongestant use and may also be used to treat different medical conditions such as dry eye disease and thus reducing the number of devices needed on a flight.

With the projected increase in private spaceflight, less stringent astronaut selection criteria will likely become more common (Griko et al., 2022). This could include candidates with preexisting allergic and chronic rhinosinusitis. These astronauts might require more aggressive treatment in orbit (oral medication, etc.) or even surgery prior to the mission to reduce risks of infections (Fokkens et al., 2020).

While there are multiple neurostimulators commercially available, few seem suitable for human spaceflight. Unlimited shelf life, rechargeability, lack of consumables and potential to be used by multiple users are crucial characteristics to be met. Despite these attractive features, they must be tested in space to develop protocols regarding duration, intensity, and use frequency because these might differ from those that are established on Earth. Chiefly, it must be determined whether neurostimulation alone is able to overcome the CFS-related increased fluid pressures. Preliminary studies during parabolic flights and short-duration spaceflights will provide insights.
